# Longitudinal Association of Salaries for Medical Staff With Medical Service Utilization and Expenditure in China, 2007–2016

**DOI:** 10.3389/fpubh.2021.767541

**Published:** 2021-12-10

**Authors:** Dawei Zhu, Na Guo, Jian Wang, Stephen Nicholas, Li Chen

**Affiliations:** ^1^China Center for Health Development Studies, Peking University, Beijing, China; ^2^China Population and Development Research Center, Beijing, China; ^3^Dong Fureng Economic and Social Development School, Wuhan University, Beijing, China; ^4^School of Economics and School of Management, Tianjin Normal University, Tianjin, China; ^5^Australian National Institute of Management and Commerce, Eveleigh, NSW, Australia; ^6^Research Institute for International Strategies, Guangdong University of Foreign Studies, Guangzhou, China; ^7^Newcastle Business School, University of Newcastle, Newcastle, NSW, Australia; ^8^Georgia Prevention Institute, Department of Population Health Sciences, Medical College of Georgia, Augusta University, Augusta, GA, United States

**Keywords:** medical service utilization, medical expenditure, salaries, physician-induced demand, China

## Abstract

**Objectives:** To test the hypothesis that higher salary levels of the medical staff are associated with lower medical service utilization and expenditure.

**Methods:** Using longitudinal data from 31 Chinese provinces for the period 2007-2016, we constructed fixed effects models to analyze the association between the salary of medical staff and medical service utilization, medical expenditure, medication expenditure, and medication proportion.

**Results:** A 10,000 CNY increase in medical staff's salaries was associated with a 0.89% decrease in the average number of annual inpatient admissions per person; 1.88 and 1.59% decreases in average expenditures per outpatient visit and inpatient admission, respectively; 3.05 and 2.66% decreases in drug expenditures per outpatient visit and inpatient admission, respectively; 0.58 percent point and 0.39 percent point decreases in the share of drug expenditure in outpatient and inpatient, respectively. When medical staff's salaries increased by 450,000 CNY, the turning point was reached when the maximum medical expenditure savings offset the medical staff salary increases, yielding a 634 billion CNY surplus from medical expenditure.

**Conclusions:** Our results supported the hypothesis that higher salary levels of the medical staff are associated with lower medical service utilization and expenditure. Further studies are requested to test whether higher medical staff's salaries will attenuate over-treatment and that savings from reduced prescriptions and service charges will offset the increased salaries of medical staff.

## Introduction

Containing health care costs is a critical challenge for China's health system. Between 2010 and 2015, per capita health care expenditure grew by 14.7 percent annually outpacing the 11.2 percent growth rate of per capita gross domestic product (GDP) (1). Over-treatment, particularly over-use of medications with no positive health outcomes, has been identified as the major component of China's health expenditure (2). Previous studies have revealed that half of all Chinese health care expenditure is accounted for medication payments (3), with this percentage as high as 70-80% in some areas (4, 5). One of the major reasons for over-treatment is the compensation system, which allows the hospital to acquire benefits from drug by around 15% mark-ups on sale prices (6). The compensation setting creates financial incentives for physicians to over-prescribe and greatly aggravates the financial burden on both the government and patients.

Approaches to addressing these issues have focused mainly on how to break the chain linking physicians' pay to drug prescriptions, such as the separation of prescribing and dispensing (7), zero mark-ups on pharmaceutical prices (8–11) and reform of the payment system (12). However, these solutions remain unsuccessful (13). It is an urgent need to identify effective approaches that would able to reduce over-treatment and contain the growth rate of medical expenditure. A recent review suggested that physicians' income was an important factor that directly influences physician-induced demand (PID) (14). Low income would provide incentives to compensate for this reduction via inducing demand (14). Enlighted by this, in this study, we aim to test the hypothesis that higher salary levels of the medical staff are associated with lower PID. Therefore, we tested the hypothesis by examining the association between salary of medical staff and health care expenditure using ten-year panel data from all 31 provinces across China.

## Methods

### Data Sources

We generated the study data by the integration of two official sets of 10-year statistical yearbooks. First, the China Statistical Yearbook, published by the National Bureau of Statistics of the People's Republic of China (15), provided social and economic factors as control variables, such as GDP per capita, age structure of population, average salary of employed person and illiteracy rate of each province. Second, China Health and Family Planning Statistical Yearbook, published by the National Health Commission of the People's Republic of China yielded data about health care resources, utilization, and health expenditure. All the variables were collected at province-level for the years 2007-2016, except for average salaries of medical staff, which were obtained for 2006-2015 for the reason explained below. The final statistical database included 310 observations comprising 31 regions (simplified as provinces) for 10 panels.

### Medical Service Utilization and Expenditure Measurements

We used the average annual number of outpatient visits and inpatient admissions per person to measure the utilization of health care service. The main measures of health care expenditure were average expenditures per outpatient visit and per inpatient admission. Since medication over-prescribing is a significant component of unnecessary health care spending, drug expenditures per outpatient visit and per inpatient admission were also used as indicators of over-prescribing profit-seeking behavior. In addition, the shares of drug expenditure in outpatient and inpatient were used for that with economic growth, medication expenditure might grow steadily even without physician over-treatment.

### Measurement and Calculation of Medical Staff Salary

The key explanatory variable was the previous year's relative salary of medical staff, which is used as a proxy indicator of physician's salaries, which were not included in the yearbooks, since the salaries of medical staff and physicians are highly correlated. The pairwise correlation coefficient of salaries of medical staff and physicians in 2016 was 0.94 (*p*-value < 0.001). Salaries were reported by each hospital and summarized by local governments. Salaries do not include any remuneration received from medical representatives or pharmaceutical companies, the so-called “gray income.” We assume that if physicians can get a satisfactory level of income just from their salary, then the motivation to acquire extra benefits from over-treatment and “gray income” would be attenuated. The “gray income” is the outcome of underpayment, not the cause. Therefore, the explanatory income variable was formal income only. Considering that medical expenditure per patient may have a positive impact on physician salaries in the same time period, we applied the previous year's salary as an explanatory variable to calculate its relation with the outcome variables, such as medical expenditures, which may avoid the reciprocal causation problem. Therefore, the panel study used salary data from the year 2006-2015 to indicate its relationship with outcome variables in 2007-2016 period. As income satisfaction is affected by relative income, we used the difference between the salary of medical staff and local employed persons instead of absolute income (16).

### Statistical Analysis

This study uses longitudinal dataset to assess the relationships between the dependent and independent variables over a 10-year period. To offset potential problems associated with omitted variable biases, fixed effects (FE) model was employed. An advantage of the fixed effects model is that it controls for time-invariant heterogeneity among provinces, such as underlying aspects of local culture and so on. To control for recent health reforms, time dummies were included to control aggregate time-specific effects that may affect health care quantity, which would not be captured by province explanatory variables. Logarithmic transformations were performed on per capita GDP and all outcome variables except the share of drug expenditure in outpatient and inpatient to adjust for the skewed monetary variables. The regression models also included macro-level determinants, including GDP per capita, practicing physicians per thousand population, dependency ratio (proportion of population under 14 and over 65), and illiteracy rate, as covariates. GDP per capita was used to control for province economic development differences and yearly price differences, while practicing physicians per thousand population was used to account for the unbalanced health care resources among areas. The illiteracy rate reflected overall province educational status, and dependency ratio was utilized to capture the population age structure. A *p*-value of < 0.05 was considered statistically significant. The software Stata version 15 for Windows (Stata Corp, College Station, TX, USA) was used for the statistical analysis.

## Results

### Descriptive Results

Table 1 summarizes our variables for 31 Chinese provinces. During the period from 2007 to 2016, the average number of outpatient visits and inpatient admissions per person per year was 1.90 and 0.09, respectively, and the average expenditure per outpatient visits and inpatient admission were 176.93 CNY and 6922.31, respectively. The share of drug expenditure in outpatient was 47.49% which was a little higher than that in inpatient (40.84%). The salary of medical staff was about 74,000 CNY, which was 27 thousand more than that of local employed persons.

**Table 1:** Basic characteristics of 31 provinces of China, 2007-2016 (*N* = 310).

**Characteristic**	**Mean**	**SD**
**Dependent variables**		
Average number of outpatient visits per person per year	1.90	1.18
Average number of inpatient admissions per person per year	0.09	0.03
Average expenditures per outpatient visit	176.93	61.82
Average expenditures per inpatient admission	6922.31	3005.69
Drug expenditures per outpatient visit	85.17	37.59
Drug expenditures per inpatient admission	2770.01	1029.93
The share of drug expenditure in outpatient (%)	47.49	5.76
The share of drug expenditure in inpatient (%)	40.84	4.58
**Independent variable**		
Previous year relative salary of medical staff (10,000 RMB)	2.69	1.91
**Control variables**		
GDP per capita (10,000 RMB)	4.00	2.24
Density of practicing physicians per thousand population	5.16	1.87
Dependency ratio	35.68	6.48
Illiteracy rate	7.31	6.28

### Association of Salary With Health Care Utilization and Expenditure

Fixed effects regression results are shown in Tables 2, 3. In each of the outcome variables presented, two nested models were compared. The first model (model 1) contains only previous year's relative salary of medical staff. The second model (model 2) contains previous year relative salaries of medical staff in addition to macro-level factors (GDP per capita, practicing physicians per thousand population, dependency ratio, and illiteracy rate). All models adjusted for province and yearly fixed effects.

**Table 2:** Adjusted association between previous year relative salary and medical service utilization and expenditure.

	**Log average number of annual outpatient visits per person**		**Log average number of annual inpatient admissions per person**		**Log average expenditures per outpatient visit**		**Log average expenditures per inpatient admission**	
	**Model 1**	**Model 2**	**Model 1**	**Model 2**	**Model 1**	**Model 2**	**Model 1**	**Model 2**
Previous year relative salary	−0.010^*^	−0.004	−0.022^***^	−0.009^*^	−0.024^***^	−0.019^***^	−0.016^***^	−0.016^***^
	(0.004)	(0.003)	(0.005)	(0.004)	(0.004)	(0.004)	(0.004)	(0.004)
Control variables	No	Yes	No	Yes	No	Yes	No	Yes
Province fixed effects	Yes	Yes	Yes	Yes	Yes	Yes	Yes	Yes
Yearly fixed effects	Yes	Yes	Yes	Yes	Yes	Yes	Yes	Yes
R-square	0.940	0.955	0.946	0.974	0.947	0.954	0.947	0.951
Observations	310	310	310	310	310	310	310	310

*Covariates include practicing physicians per thousand population, GDP per capita, dependency ratio, and illiteracy rate*.

* *p < 0.05*,

** *p < 0.01*,

*** *p < 0.001*.

**Table 3:** Adjusted association between previous year relative salary and drug expenditures.

	**Log drug expenditures per outpatient visit**		**Log drug expenditures per inpatient admission**		**The share of drug expenditure in outpatient (%)**		**The share of drug expenditure in inpatient (%)**	
	**Model 1**	**Model 2**	**Model 1**	**Model 2**	**Model 1**	**Model 2**	**Model 1**	**Model 2**
Previous year relative salary	−0.033^***^	−0.031^***^	−0.026^***^	−0.027^***^	−0.467^***^	−0.577^***^	−0.331^**^	−0.385^***^
	(0.005)	(0.005)	(0.005)	(0.005)	(0.118)	(0.122)	(0.105)	(0.108)
Control variables	No	Yes	No	Yes	No	Yes	No	Yes
Province fixed effects	Yes	Yes	Yes	Yes	Yes	Yes	Yes	Yes
Yearly fixed effects	Yes	Yes	Yes	Yes	Yes	Yes	Yes	Yes
R-square	0.905	0.911	0.838	0.852	0.419	0.443	0.779	0.788
Observations	310	310	310	310	310	310	310	310

*Covariates include practicing physicians per thousand population, GDP per capita, dependency ratio, and illiteracy rate*.

* *p < 0.05*,

** *p < 0.01*,

*** *p < 0.001*.

The results show that medical staff salary is negatively associated with the average number of annual outpatient visits per person independently (model 1), but it is not statistically significant in full model (model 2) that includes macro-level determinants. Medical staff salary is negatively associated with the average number of annual inpatient admissions per person, average expenditures per outpatient visit and average expenditures per inpatient admission in all models, and is statistically significant (*P* < 0.05). The effect of medical staff salary is partially reduced in the presence of macro-level determinants. In fully adjusted models (model 2), an increase of 10,000 CNY in medical staff salary was associated with a 0.89% (1 – e^∧^−0.009) decrease in the average number of annual inpatient admissions per person, a 1.88% (1 – e^∧^−0.019) decrease in average expenditures per outpatient visit, and a 1.59% (1 – e^∧^−0.016) decrease in average expenditures per inpatient admission, respectively.

Table 3 presents the results for drug expenditure and its' share. Medical staff salary is negatively associated with all outcomes in all models. The effect of medical staff salary is partially increased in the presence of macro-level determinants except for drug expenditures per outpatient visit. In fully adjusted models (model 2), an increase of 10,000 CNY in medical staff salary was associated with a 3.05% (1 – e^∧^−0.031) decrease in drug expenditures per outpatient visit, a 2.66%(1 – e^∧^−0.027) decrease in drug expenditures per inpatient admission, a 0.58 percent point decrease in the share of drug expenditure in outpatient, and a 0.39 percent point decrease in the share of drug expenditure in inpatient, respectively.

### Predicted Impact of Salary Increase on Health Expenditures

**Figure 1:**
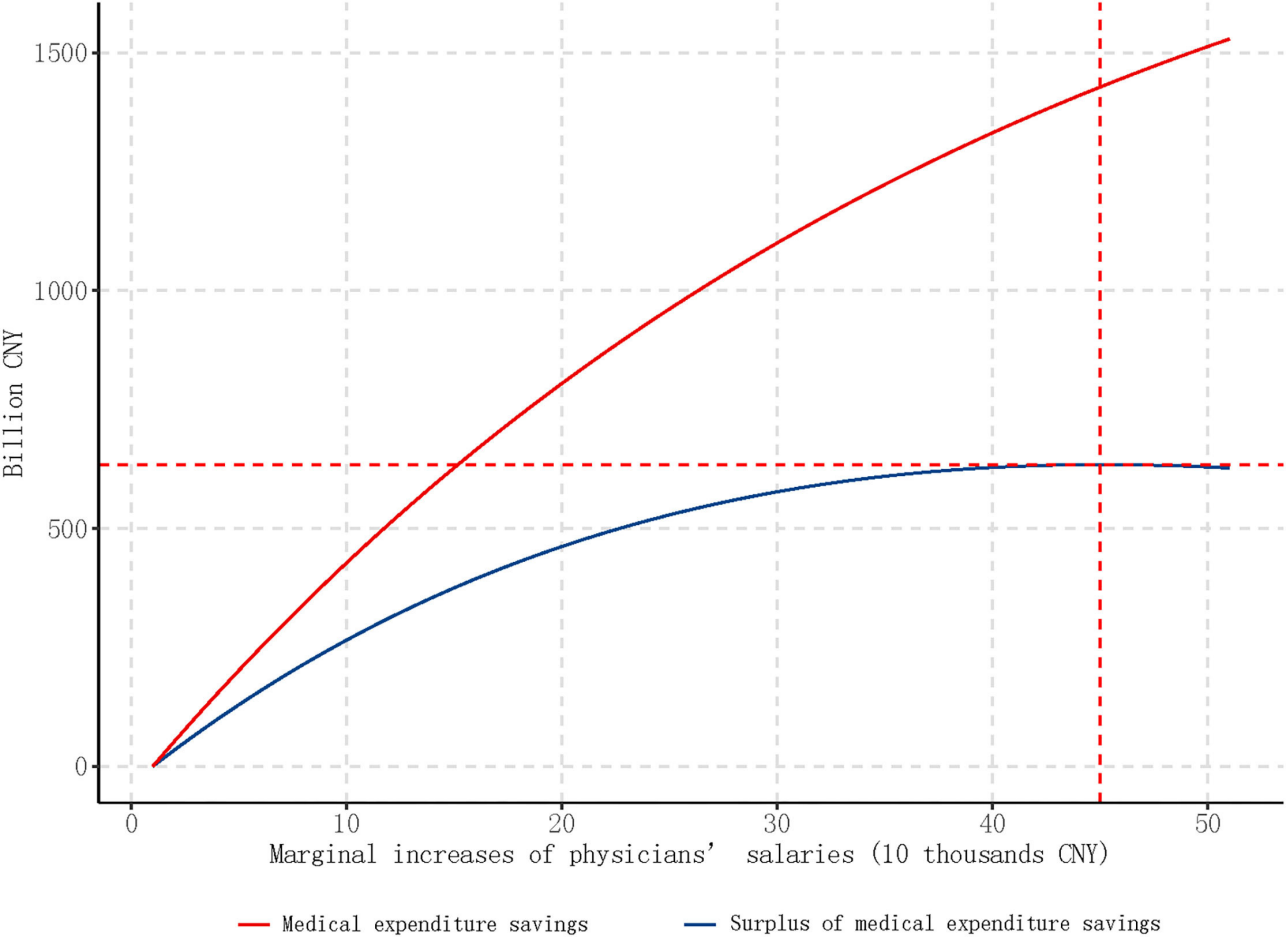
Predicted medical expenditures savings and surplus based on different marginal increases of salary. In 2016, China's population is 1.38 billion, and there are total 1,803,000 medical practitioners (assistants) in hospitals.

To further explore the association between medical staff salary and health care expenditures, we used the results displayed in Table 2 to predict the health care expenditure (Figure 1) based on different marginal increases of medical staff salaries. For example, a medical staff salary increase of 100,000 CNY is related to a decrease of 21.7% (roughly 470 billion CNY) in total outpatient and inpatient expenditure. Importantly, the total medication expenditure saved was greater than the medical staff salary increases. As shown in Figure 1, the turning point offsetting the maximum medical expenditure savings against medical staff salary increases was 450,000 CNY (about 65,000 USD), yielding a 634 billion CNY surplus of health care expenditure savings.

## Discussion

Our study shows that medication expenditure, medical expenditure, and medication proportion are all negatively related to the medical staff's previous year relative salary. The results support the hypothesis that higher medical staff's salary is associated with lower medical expenditure. Moreover, our findings show that the additional costs of increased medical staff salaries are more than offset by the savings from reduced over-servicing expenses.

Based on our findings, we cautiously propose the possibility that raising physicians' salaries may offer an effective cost-containment mechanism for arresting rising health costs due to over-treatment. Our models showed that 450,000 CNY (about 65,000 USD) increases of the medical staff annual salary, which means a total of 812 billion CNY salary increase nationwide, were associated with a total of 1,446 billion CNY decreases of medical expenditure. Moreover, we identified an inverted U-shape net saved expenditure-salary increase curve, with the optimal increased salary cutoff point at 450,000 CNY (about 65,000 USD).

A key insight from the negative relationship between the medical staff's salary and health care cost is the law of diminishing marginal utility (17). There are costs when a physician acquires extra income by over-treatment, including the risk of being caught and punished and damage to one's reputation as an honest physician (18). According to the law of diminishing marginal utility, each incremental income increase in a physicians' annual salary yields less utility. Since at higher salary levels the difference in utility from each additional unit extra income will be smaller than at lower income levels, the motivation of earning extra money through over-prescribing drugs or over-servicing will be weaker at higher physician salaries, especially when there is effective monitoring of over-treatment.

According to our data, the average annual salary of the medical staff in China is 74,000 CNY (about 11,000 USD), which is low in contrast to physicians' high training costs, income foregone during the study and training, and deteriorating working environment, such as violence toward physicians (19). Such low salary provides the physicians with great incentives to seek additional income, which probably include over-treatment (20–23). Such PID is a nation-wide phenomenon, but with salaries in rural areas especially low, we expect that PID could be a persistent problem in rural and relatively economically underdeveloped regions. Our modeling suggests the possibility that when physicians' salaries rise, over-treatment may decline.

One implication of this study is that if the current salary situation remains unchanged, regulations from government departments will become passive. Physicians have the incentives to actively seek higher incomes by using their superior information advantage relative to government departments. Although government price regulation policy can restrict over-treatment, the physicians still have scope to over-prescribe medications, over-service patients and benefit from secret contracts with pharmaceutical companies, a form of “gray income” (24). On the contrary, higher salaries could lower the motivation to acquire additional income, especially “gray income,” diminishing distortions to China's health care costs. Our results suggest that raising physician salaries could be an effective and efficient policy response to China's health care costs. However, more evidence is needed to support this hypothesis.

To the best of our knowledge, we are the first to identify an inverse relationship between the medical staff salaries and medical expenditure over time in China. This study included 10-year statistics from 31 Chinese provinces, which can establish the longitudinal association of medical staff salary with medical service utilization and expenditure. Moreover, our results provide preliminary evidence that increasing the salaries of medical staff could be an effective intervention to contain costs related to the provision of unnecessary health care, with higher salaries more than offset by savings in health care costs. However, our research was also subject to several limitations. First, our analyses were appropriately ecological, and province-level data cannot fully reflect individual preference. However, provincial-level aggregated data has been widely applied in public health research (25–28). Further, individual salary and prescription data are both highly sensitive and restricted in China. Although we encourage researchers to collect individual data to test our hypothesis, we note the difficulties in collecting highly sensitive individual data, which might result in extremely limited, and likely highly biased data sets. Given the absence of individual data, and the challenges of collecting such data, province-level aggregated data on physician salaries are the most robust of the currently available data (29). Secondly, medical expenditure of each province is influenced by factors other than physician behavior, including health condition of residents, health policy, insurance coverage and availability of medication. In the regression model, we have mitigated this problem by collecting variables, such as proportion of out-of-pocket expenditure and density of practicing physicians per thousand of the population. In addition, our panel data study design which adjusted province and yearly fixed effects has also addressed this issue and was able to control for those confounding factors. Further studies are required to replicate our findings.

## Conclusions

Our results support the hypothesis that higher salary levels of the medical staff are associated with lower medical service utilization and expenditure. Our findings indicate that higher medical staff salaries might attenuate over-treatment and that savings from reduced prescriptions and service charges may offset the increased salaries.

## Data Availability Statement

The raw data supporting the conclusions of this article will be made available by the authors, without undue reservation.

## Author Contributions

DZ and LC participated in the design of the study, performed the statistical analysis, and drafted the manuscript. NG contributed to conduct the study, collect research data, figures, and paper preparation. JW participated in the interpretation of data and revised the paper. SN was involved in drafting the manuscript and revision. All authors read and approved the final manuscript.

## Conflict of Interest

The authors declare that the research was conducted in the absence of any commercial or financial relationships that could be construed as a potential conflict of interest.

## Publisher's Note

All claims expressed in this article are solely those of the authors and do not necessarily represent those of their affiliated organizations, or those of the publisher, the editors and the reviewers. Any product that may be evaluated in this article, or claim that may be made by its manufacturer, is not guaranteed or endorsed by the publisher.
